# Personality Traits in Burning Mouth Syndrome Patients With and Without a History of Depression

**DOI:** 10.3389/fpsyt.2021.659245

**Published:** 2021-07-29

**Authors:** Trang Thi Huyen Tu, Motoko Watanabe, Takayuki Suga, Chaoli Hong, Chihiro Takao, Miho Takenoshita, Haruhiko Motomura, Akira Toyofuku

**Affiliations:** Department of Psychosomatic Dentistry, Graduate School of Medical and Dental Sciences, Tokyo Medical and Dental University, Tokyo, Japan

**Keywords:** big five traits, neuroticism, burning mouth syndrome, depression, catastrophizing, central sensitization

## Abstract

**Objectives:** So far, the strong link between neuroticism, chronic pain, and depression has been well-documented in literatures. Some suggested that they might share etiological factors, thus resulting in overlapping constructs. However, such effect has never been tested in burning mouth syndrome (BMS) patients, a complex phenomenon influenced by both neuropathic and psychopathological factors. We aim to clarify how personality affects individual's pain and pain-related experiences.

**Methods:** Two hundred forty-eight patients with BMS provided demographic information and psychiatric history; completed Ten-Item Personality Inventory, a Visual Analog Scale of pain, and McGill Pain Questionnaire; and provided adequate parameters of depressive state, catastrophizing thinking, and central sensitization.

**Results:** BMS patients with depression history suffered more severe clinical symptoms and scored higher in neuroticism and less in openness and extraversion than did those without psychiatric diagnoses. After age, sex, and duration of pain were controlled, neuroticism in BMS patients with depression correlates with affective dimension of pain. Instead, if psychiatric history is absent, neuroticism correlates with sensory dimension and pain intensity. In both groups, higher neuroticism, unlike other personality facets, contributed to a more severe clinical condition.

**Conclusion:** Of the five traits, neuroticism appears to be the most crucial dimension associated with the pain symptoms and patient's conditions. This study implies that management of pain must extend beyond solely providing pain-relieving medication and must require a holistic and multidisciplinary approach.

## Introduction

Burning mouth syndrome (BMS) is referred to as the most well-documented chronic oral pain. Its origin is largely multifactorial, with clinical symptoms ranging from simple burning pain of tongue to uncomfortable dysesthesia of other oral mucosal without evident signs (1, 2). Given the fact that BMS is a complex-to-handle, subjective experience, there lacks a gold standard in diagnosis and treatment. To date, neither pharmacotherapy nor psychotherapy could benefit all BMS patients. In a recent review, we discussed the efficacy of central neuromodulators in BMS management and proposed the involvement of central sensitization, which is characterized by the hypersensitivity to stimuli, as a consequence of neural dysregulation and hyper-excitability in the central nervous system (2). This condition was theorized as the root pathophysiological mechanism of non-organic subgroup of functional somatic syndromes (e.g., fibromyalgia, irritable bowel syndrome, and nonspecific chronic low back pain), which may share common etiology with BMS (3).

In terms of personality, this multifaceted combination reflects individual differences in thoughts, behaviors, and ability to cope with life stressful events. Whereas, previous studies suggest a relation between specific pain-prone personality characterized by a high level of neuroticism [one of the five factors in the five-factors model (FFM)] and BMS, its effects on pain perception are still controversial (4–7). There also remains a question of whether the relationship between personality and pain experiences in BMS is causality or merely correlation (8). While Grushka et al. suggested that personality disturbances tend to increase with a higher level of pain (7), other researchers theorize that certain personality traits predispose the development of chronic pain syndromes and contribute to how a person perceives and appraises pain (9, 10). In particular, a high neurotic person has a lower pain threshold and higher tendency to catastrophize (11). Notably, existing research on BMS tends to signify the role of psychopathological aspects rather than focus on personality and cognitive-affective constructs in this population (4).

Among psychiatric comorbidities in BMS, depression is the most common, with reported prevalence from 27.2 to 56.7% (12, 13). So far, the strong-linked relationship between neuroticism, pain, and chronic depression has been well-documented. Some suggested that they might share etiological factors, thus resulting in overlapping constructs (14, 15). Other studies argue that depressive symptoms might increase neuroticism and decrease extraversion, mainly at small and limited state effects (16, 17). Indeed, neuroticism is said to have biological origin, to mature until early adulthood, and then remain relatively consistent over time (especially in comparison with other psychological distress like depression or anxiety) (18–20). Altogether, it raises the question of how personality, pain, and depression interact. Current literature does not show us a clear picture, and yet such kind of effect has never been tested in BMS patients, particularly between patients with and without depression comorbidity. The analysis of this complex intertwining framework is therefore critical to the implication of treatment, by identifying at-risk individuals and tailoring approaches.

Hence, our objectives were as follows: (1) compare pain characteristics, depressive state, pain-related catastrophizing, central sensitization, and personality features among BMS patients with and without a history of depression; (2) determine the extent to which personality traits influence pain (intensity, component of sensory, or affective), depressive state, pain-related catastrophizing, and central sensitization.

## Methods

### Procedures and Participants

Participants of the study were recruited among patients who visited the Psychosomatic Dentistry Clinic at Tokyo Medical and Dental University in Tokyo, Japan, for the first time between July 2018 and April 2020. Inclusion criteria were as follows: (1) age over 18; (2) had no difficulty communicating in Japanese; and (3) had a diagnosis of BMS. The diagnosis was based on the definition of “a chronic intraoral burning sensation that has no identifiable cause either local or systemic condition or disease,” proposed by the International Association for the Study of Pain (21). The unexplained nature of burning symptoms was confirmed by a comprehensive step-by-step examination and necessary laboratory tests. Patients were excluded if they had conditions that hinder reliable data collection (e.g., neurodevelopmental disorder, dementia, and difficulty in reading, understanding, and/or answering questions). Informed written consent was obtained from all the patients, and the study protocol was approved by the Ethical Committee of Tokyo Medical and Dental University (D2013-005).

### Parameters

The following sociodemographic information was collected: age, sex, and duration of illness. Psychiatric history was obtained from referral letters from the patients' psychiatrists. At the first visit, we required all patients to submit referrals if they had experienced any history of psychiatric disorder. The diagnosis was then adopted as given by their attending psychiatrists. In case the patients reported such history but could not provide referrals (for any reason), we then directly contacted their psychiatrists to inquire further details. Patients with different psychiatric diagnoses other than depression (depressive disorders, *Diagnostic and Statistical Manual of Mental Disorders*, 5th ed.) were then excluded, leaving the final two groups of BMS patients: one with a history of depression and the other without any history of psychiatric disorder.

In this present study, we assessed the personality of BMS patients using the FFM (22). This framework comprises extraversion, agreeableness, conscientiousness, neuroticism, and openness. FFM is one of the most used and studied models of personality. All patients answered the Japanese version of the Ten-Item Personality Inventory (TIPI-J), a concise validated questionnaire of FFM. Each of the 10 items was rated from 1 (strongly disagree) to 7 (strongly agree) (23).

The pain characteristics were examined using the short form of the McGill Pain Questionnaire (SF-MPQ) (24). It contains 15 descriptors: 1 to 11 represent the sensory dimension, and 12 to 15 represent the affective dimension. The Visual Analog Scale (VAS) indicating pain severity was 10 cm in length and anchored by 0 (no burning pain) and 100 (worst pain imaginable).

Patients also completed a 20-item assessment tool known as Zung's Self-Rating Depression Scale (SDS) for the current depressive state (25). Responses range from 1 (“a little of the time”) to 4 (“most of the time”), and the total score ranges between 20 and 80.

Patients' feelings and attitudes toward the painful situation (i.e., the tendency toward rumination, magnification, and helplessness due to BMS) were examined using the Pain Catastrophizing Scale (PCS). Patients answered 13 items in total, each of which is rated on a 5-point Likert scale (from 0 “not at all” to 4 “all the time”) (26). In total, score ranges from 0 to 52.

To assess central sensitization severity levels in patients with BMS, we used a high reliability and validity screening instrument named Central Sensitization Inventory (CSI) (27). It consists of 25 questions on overlapping health-related symptom dimension of central sensitization, and patients are to answer using a scale from 0 (never) to 4 (always). A cumulative score ranges between 0 and 100.

### Statistical Analysis

The statistical analysis was performed using R version 3.4.2 for Mac OS (R Foundation for Statistical Computing, Vienna, Austria). Probability values of *p* <0.05 were accepted as statistically significant. Missing values were imputed (“MICE” package, R Foundation for Statistical Computing) in accordance with the precondition that they comprise <5% of total dataset and are random. We applied the chi-square test and the Kruskal–Wallis test to compare clinical characteristics (pain characteristics, depressive state, and pain-related catastrophizing), central sensitization severity level, and personality between the two BMS patient groups. A correlation matrix of Kendall's rank coefficients τ showing pairwise associations between personality and clinical characteristics was built. All the *p*-values were then adjusted for multiple tests, using the Holm–Bonferroni method (28). Afterward, we performed multivariate linear regression models to measure the degree of the associations between each personality dimension and a series of clinical characteristics (controlling for covariates: age, sex, duration of illness). In each model, one of five TIPI traits is the independent factor with age, sex, and illness duration, while six clinical parameters are the dependent variables. This model entails an additional variance–covariance matrix of the model coefficients. This helps estimate whether an independent variable jointly contributed to multiple dependent variables and also represents multicollinearity if the variables are highly correlated. Subsequently, the regression validation is conducted visually via regression diagnostic graphs, meaning evaluating the regression assumptions (e.g., the residuals are normally distributed, and their variance does not change as a function of X).

## Results

### Baseline Characteristics

Data of 343 patients were reviewed. Ten patients were excluded due to conditions that could affect the reliability of their self-reports (schizophrenia, 4; dementia, 1; Alzheimer's disease, 2; mild cognitive impairment, 1; intellectual disability, 1; and Parkinson's disease, 1). Eighty-five with history of psychiatric disorders other than depression were omitted (anxiety disorders, 19; insomnia disorders, 18; somatic symptom and related disorders, 15; bipolar and related disorders, 3; obsessive-compulsive disorders, 1; unknown, 38). After the application of all criteria, data for 248 patients were available for analysis. The patients' mean age was 62.07 ± 13.53 years, and 81.5% of them were female. Patients had experienced pain for average 35.56 ± 46.63 months. The demographic characteristics, comorbid psychiatric disorders, and symptom-related parameters of 248 patients with BMS are shown in Table 1. Of 248 patients, 55 (22.2%) have experienced visiting a psychiatrist and were diagnosed as having major depressive disorder (90.9%) or dysthymia (9.1%).

**Table 1:** Demographic characteristics and symptom-related parameters of 248 BMS patients.

	***N* (%) or Mean ± SD**
Female	202 (81.5)
Age (year)	62.07 ± 13.53
Duration of symptom (month)	35.56 ± 46.63
No psychiatric history	193 (77.8)
Depressive disorders	55 (22.2)
Major depression disorder	50 (90.9)
Dysthymia	5 (9.1)
SF-McGill Pain Characteristics	
Sensory dimension	2.58 ± 2.98
Affective dimension	4.62 ± 4.9
Pain intensity (VAS)	48.34 ± 27.7
Depressive state (SDS)	42.56 ± 10.7
Catastrophizing thinking (PCS)	28.33 ± 11.88
Central sensitization (CSI)	24.36 ± 14.22

*SD, Standard Deviation; VAS, Visual Analog Scale; SDS, Self-Depression Scale; PCS, Pain-related Catastrophizing Scale; CSI, Central Sensitization Inventory*.

### Comparison of Clinical Characteristics, Central Sensitization Level, and Personality Traits by the Presence of Depression History

**Figure 1:**
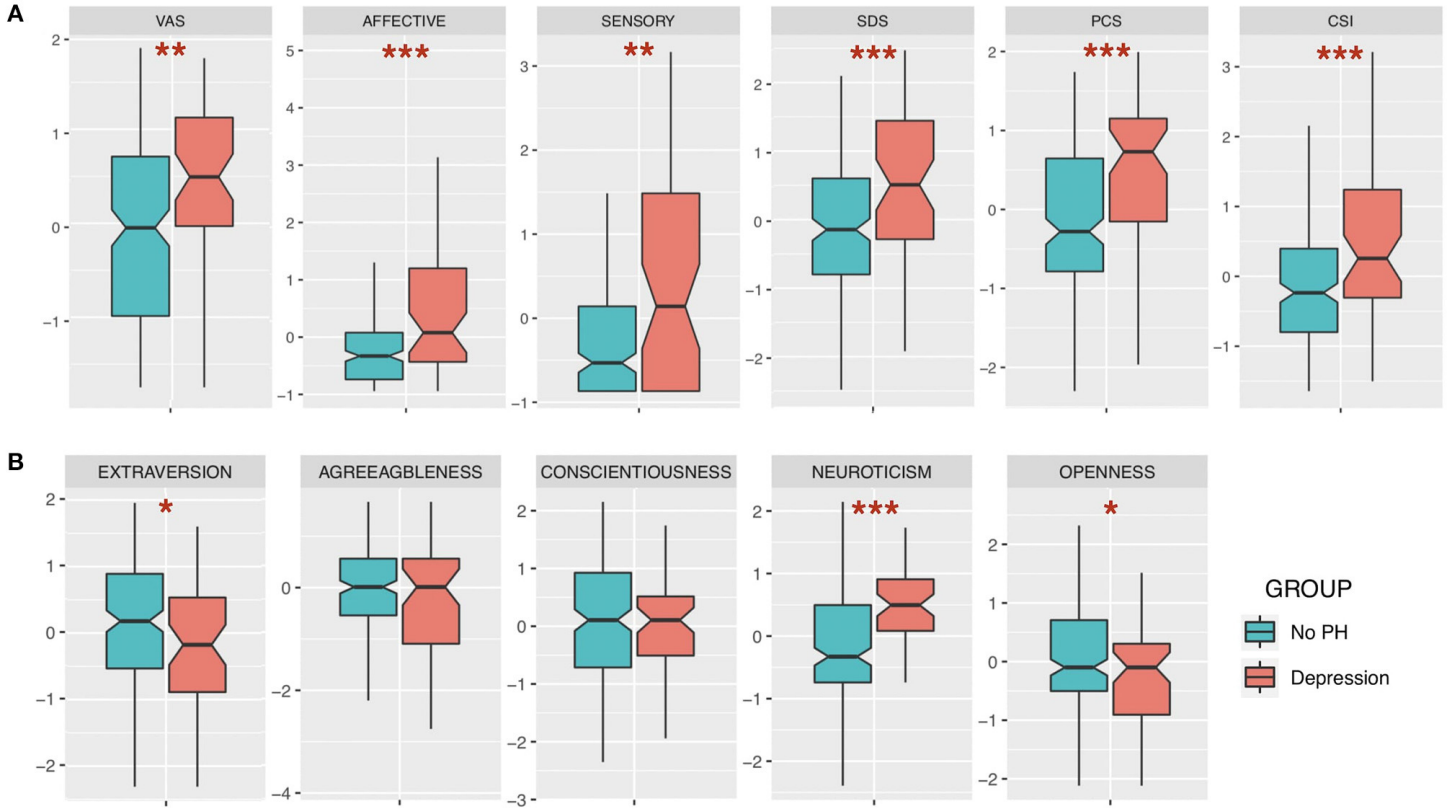
Comparisons between groups of burning mouth syndrome (BMS) patients with depression history and without psychiatric history (standardized). **(A)** Clinical measures of pain, pain characteristics, depressive state, catastrophizing, and central sensitization. **(B)** Big five traits. VAS, Visual Analog Scale; SDS, Self-Rating Depression Scale; PCS, Pain-related Catastrophizing Scale; CSI, Central Sensitization Inventory. Wilcoxon rank sum test; *p < 0.05; **p < 0.01; ***p < 0.001.

Despite no significant differences in demographic data (age, sex, and duration of illness: all *p*-values > 0.05), BMS patients with history of depression score higher in every aspect of clinical characteristics (pain, depressive state, and catastrophizing) and central sensitization level than those without psychiatric history (Figure 1A). Regarding the big five traits, the BMS group with history of depression had a significantly higher score in neuroticism (p < 0.001), but a lower score in openness and extraversion (*p* < 0.05). Other traits had differences, but no statistical significance was detected (Figure 1B). As seen in Table 2, there are different significantly positive correlations between scores of VAS, sensory, affective component of SF-MPQ, and SDS, PCS, and CSI in each group. However, all τ coefficients were higher in the group of patients with depression comorbidity.

**Table 2:** Correlations between measures (unstandardized, Kendall's rank correlation *tau, P-values* were adjusted for multiple tests).

	**E**	**A**	**C**	**N**	**O**	**VAS**	**Affective**	**Sensory**	**SDS**	**PCS**	**CSI**
**(A) BMS patients without psychiatric history**											
1. Extraversion		0.13	0.13	−0.16	0.19	0.00	−0.01	−0.04	−0.10	−0.05	−0.06
2. Agreeableness			0.28^**^	−0.21	−0.01	0.04	0.06	0.05	−0.21	0.02	−0.16
3. Conscientiousness				−0.22	0.09	0.01	0.07	0.07	−0.14	−0.02	−0.05
4. Neuroticism					−0.16	0.11	0.09	0.09	0.31^***^	0.22	0.26^*^
5. Openness						−0.01	0.08	−0.01	−0.15	−0.07	0.01
6. VAS							0.40^***^	0.35^***^	0.14	0.24^*^	0.15
7. Affective								0.43^***^	0.14	0.28^**^	0.23
8. Sensory									0.22	0.35^***^	0.22
9. SDS										0.29^***^	0.34^***^
10. PCS											0.31^***^
11. CSI											
**(B) BMS patients with a history of depression**											
1. Extraversion		0.03	0.21	−0.12	0.27	−0.15	−0.11	−0.14	−0.20	−0.20	−0.24
2. Agreeableness			0.34	−0.21	−0.16	0.02	−0.13	−0.12	−0.10	−0.11	−0.24
3. Conscientiousness				−0.25	−0.09	−0.08	−0.13	−0.15	−0.11	−0.23	−0.22
4. Neuroticism					−0.03	0.05	0.29	0.08	0.20	0.30	0.19
5. Openness						−0.15	−0.03	−0.03	−0.20	−0.03	0.03
6. VAS							0.47^*^	0.52^**^	0.25	0.39	0.26
7. Affective								0.54^**^	0.21	0.40	0.39
8. Sensory									0.22	0.47^*^	0.33
9. SDS										0.42	0.49^**^
10. PCS											0.46^*^
11. CSI											

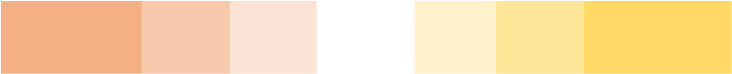

*−1 −0.4 −0.2 −0.1 0 0.1 0.2 0.4 1.*

*VAS, Visual Analog Scale; SDS, Self-Depression Scale; PCS, Pain-related Catastrophizing Scale; CSI, Central Sensitization Inventory.*

* *P < 0.05;*

** *P < 0.01;*

*** *P < 0.001 (adjusted with Holm-Bonferroni method)*.

### The Associations Between Personality Traits and Pain, Depressive State, Pain-Related Catastrophizing, and Central Sensitization Level

In group of BMS patients without psychiatric history, after sex, age, and duration of illness were controlled, significant associations were found between the following: neuroticism and sensory component of SF-MPQ (β = 0.24, *p* < 0.01, adj R^2^ = 0.053, RMSE = 2.53), VAS (β = 1.70, *p* < 0.05, adj R^2^ = 0.04, RMSE = 26.71), SDS (β = 1.75, *p* < 0.001, adj R^2^ = 0.164, RMSE = 8.88), PCS (β = 1.71, *p* < 0.001, adj R^2^ = 0.1224, RMSE = 10.77), and CSI (β = 2.09, *p* < 0.001, adj R^2^ = 0.153, RMSE = 11.95). In contrast, a higher level of agreeableness was negatively associated with SDS (β = −1.94, *p* < 0.001, adj R^2^ = 0.095, RMSE = 9.24) and CSI (β = −2.16, *p* < 0.001, adj R^2^ = 0.087, RMSE = 12.40). Interestingly, SDS is the only psychological parameter that was associated significantly with all five traits (extraversion, β = −0.57, *p* < 0.05, adj R^2^ = 0.016, RMSE = 9.63; conscientiousness, β = −0.80, *p* < 0.01, adj R^2^ = 0.029, RMSE = 9.57; openness, β = −0.90, *p* < 0.01, adj R^2^ = 0.040, RMSE = 9.52). None of the associations between pain characteristics and traits other than neuroticism were statistically significant. For details, see Table 3.

**Table 3:** Impacts of personality on clinical measures (controlled for age, sex and illness duration).

	**Extraversion**	**Agreeableness**	**Conscientiousness**	**Neuroticism**	**Openness**
**BMS patients without psychiatric history**					
**SF-McGill pain characteristics**					
Sensory dimension	−0.08	−0.19	0.05	0.24^**^	0.03
Affective dimension	0.02	−0.08	0.12	0.15	0.25
Pain Intensity (VAS)	−0.16	0.08	0.04	1.70^*^	0.03
Depressive state (SDS)	−0.57^*^	−1.94^***^	−0.80^**^	1.75^***^	−0.90^**^
Catastrophizing thinking (PCS)	−0.23	−0.64	−0.29	1.71^***^	−0.28
Central sensitization (CSI)	−0.61	−2.16^***^	−0.13	2.09^***^	0.11
**BMS patients with a history of depression**					
**SF-McGill pain characteristics**					
Sensory dimension	−0.25	−0.38	−0.26	0.16	−0.13
Affective dimension	−0.30	−0.24	−0.18	0.85^*^	−0.15
Pain intensity (VAS)	−2.07	0.17	−1.50	1.80	−2.41
Depressive state (SDS)	−1.12	−1.26	−1.07	1.50^*^	−1.46^*^
Catastrophizing thinking (PCS)	−0.89	−0.46	−1.07	1.50^*^	−0.31
Central sensitization (CSI)	−1.79^*^	−2.66^*^	−1.67	1.60	0.27

*VAS, Visual Analog Scale; SDS, Self-Depression Scale; PCS, Pain-related Catastrophizing Scale; CSI, Central Sensitization Inventory*.

*Multivariate linear regression;*

* *P < 0.05;*

** *P < 0.01;*

*** *P < 0.001.*

*All regression models were validated visually using regression diagnostic graphs*.

Moreover, if a history of depression is present, after covariates were adjusted, a higher score in neuroticism significantly correlates with a higher level of affective component (β = 0.85, *p* < 0.05, adj R^2^ = 0.050, RMSE = 5.44), whereas it has no relation with either sensory component of SF-MPQ or VAS (Table 3). Also, SDS and extraversion are no longer significantly correlated. So are agreeableness and conscientiousness.

If we define a higher level of VAS, SDS, PCS, and CSI as a negative impact (i.e., patients suffer from more severe pain, depressive state, and negative thinking and have higher central sensitization level), the results in parts A and B in Table 3 consistently show the opposite effects by neuroticism vs. the group of four other traits. Higher neuroticism contributes to worse patient conditions. Higher extraversion, agreeableness, conscientiousness, and openness are associated with less severe conditions, but their impacts vary.

## Discussion

Among many types of chronic pain, those without obvious pathology like BMS are the most difficult to deal with, thus inducing recurring expenses. As long as the dilemma of the origin BMS remains unsolved, the key to any successful treatment is still a puzzle. So far, this is the largest FFM-based personality observation in one population of BMS, in which we seek to address how individual traits associate with patients' clinical experiences and central sensitization level. Our main findings include (1) patients with a history of depression suffering from more severe clinical symptoms, had higher central sensitization, and scored higher in neuroticism and lower in extraversion and openness than those without psychiatric diagnosis; (2) neuroticism and the other four traits have inverse correlations with pain, depressive state, catastrophizing thinking, and central sensitization; and (3) higher neuroticism combined with a history of depression results in a higher level of affective dimension, while a higher level of sensory dimension and pain intensity is observed in those without psychiatric history.

Since depression and pain share biological pathways, their overlapping presence is commonly observed. Findings from chronic pain cohort studies assessing the impact of depression are in agreement with ours. This demonstrates a comorbidity of depression significantly exacerbating pain complaint, especially pain intensity, and greater impairment (29). A part of our finding is also consistent with previous work by Kim et al., suggesting that BMS patients with psychological problems experienced more intensive pain, higher stress-related symptoms, and more difficulties in daily life (30). However, in most of the cases, the questions often focus on estimating depression's prevalence and its impact on healthcare cost, rather than comparing pain-related experiences (31).

Despite unknown and ambiguous biological mechanism, personality is considered an independent efficient marker to predict overall health and well-being (14, 32, 33). Personality dysfunction has been also reported as “the most clinically salient problem” in patients with medically unexplained symptoms such as uncertain etiology like BMS (34). In terms of neuroticism, this trait is also referred to as emotional instability—a normal personality dimension that varies between individuals. It represents the degree to which a person interprets an ordinary life circumstance as threatening, negative, and unsafe. According to Smith et al., a person with high neuroticism profile has a lower threshold when perceiving a pain stimulus as threatening; thus, pain-related catastrophizing emerges (32). In agreement with our findings, Goubert et al. found that neuroticism significantly correlates with catastrophizing, vigilance of pain, and fear of movement (11). Moreover, due to evidence showing the influence of neuroticism on pain perception, some authors hypothesized the involvement via central sensitization. According to Woolf, there remains a question of “whether there are individuals with a higher inherited propensity for developing central sensitization than others” (35). A certain profile of personality might be the potential answer for those individual differences.

Of particular interest of this study is that in BMS patients with depression and high neuroticism score profiles, pain seems to be perceived as a more affective disturbance. In contrast, patients without psychiatric disorders have the sensory component elevated by higher neuroticism. A similar result was observed by Harkin et al., in which highly neurotic patients perceived their chronic pain as more disturbing than less neurotic group did, despite no difference in the sensation magnitude. However, no impact of psychiatric comorbidity was examined (36). Our findings could also be explained by prior fMRI study in fibromyalgia patients, illustrating that the presence of depression significantly associated with enhanced neural activities in the amygdalae and contralateral anterior insula, where the affective dimension is processed (37). Nevertheless, to understand thoroughly the “transition” impact of neuroticism on pain perception from sensory dimension to an affective one in BMS patients with depression comorbidity, a more comprehensive prospective design is necessary.

Although there exists debate on whether the effect of neuroticism is overreported, evidences supporting its negative role in chronic conditions have been more consistent than those of four other traits (11, 38–40). In our study, the other traits showed no or little impact on pain parameters; instead, they were partially correlated with depressive state, pain catastrophizing, and central sensitization through a variety of levels, but much less than neuroticism. Altogether, we suggest that the influence of personality on symptoms of BMS seems to be mainly driven by neuroticism.

### Strength and Limitation

This current study was limited in three main facets. The retrospective design without a control group is unable to define whether neuroticism affects chronic pain patients differently. Another potential bias is that the data were collected from a specific clinic of academic dental hospital where patients are likely to have more complex, multiple chronic conditions and suffer from more severe burning pain. Such sample might not represent the general population of BMS. Moreover, factors related to the history of depression (such as clinical definition, treatment, and response) were not fully addressed in the current study, which limit the result interpretation. The final concern is with regard to the low R^2^ in multivariate regression models. However, this might be expected, since a combination of one personality trait, age, gender, and duration might not predict very well the variation of clinical outcomes. Nevertheless, it should be noted that our study is the first one to assess the relationship of personality, pain, and depression, with the largest sample of patients among personality-related research on BMS so far.

## Conclusion

BMS patients with history of depression appear to have personality profiles different from those of the group without psychiatric history. Of the five traits, neuroticism appears to be the most crucial dimension associated with clinical experiences in BMS patients. Notably, patients with depression comorbidity and a high neuroticism score profile seem to perceive pain as affective disturbance. This study reinforces that management of pain must extend beyond solely providing pain-relieving medication and require a holistic and multidiscipline approach. Further study needs to address the role of personality in predicting patients' adherence and treatment outcome in a long-term follow-up.

## Data Availability Statement

The datasets presented in this article are not readily available due to ethical concerns and security requirements of patients-related data. Requests to access the datasets should be directed to Dr. Trang Thi Huyen Tu, tu.ompm@tmd.ac.jp.

## Ethics Statement

The studies involving human participants were reviewed and approved by Ethical Committee of Tokyo Medical and Dental University. The patients/participants provided their written informed consent to participate in this study. The study protocol was approved by the Ethical Committee of Tokyo Medical and Dental University (D2013-005) and conformed to the provisions of the Declaration of Helsinki. All patients had been informed about the possibility of their data being used for study purposes at their first visit and had provided written informed consent.

## Author Contributions

TT, HM, and AT were involved in study design, data collection, data analysis, and manuscript drafting. MW, TS, CH, CT, and MT contributed in data collection, results interpretation, and manuscript revision. All authors read and approved the manuscript.

## Conflict of Interest

The authors declare that the research was conducted in the absence of any commercial or financial relationships that could be construed as a potential conflict of interest.

## Publisher's Note

All claims expressed in this article are solely those of the authors and do not necessarily represent those of their affiliated organizations, or those of the publisher, the editors and the reviewers. Any product that may be evaluated in this article, or claim that may be made by its manufacturer, is not guaranteed or endorsed by the publisher.
